# Using an Adjusted Serfling Regression Model to Improve the Early Warning at the Arrival of Peak Timing of Influenza in Beijing

**DOI:** 10.1371/journal.pone.0119923

**Published:** 2015-03-10

**Authors:** Xiaoli Wang, Shuangsheng Wu, C. Raina MacIntyre, Hongbin Zhang, Weixian Shi, Xiaomin Peng, Wei Duan, Peng Yang, Yi Zhang, Quanyi Wang

**Affiliations:** 1 Beijing Center for Disease Prevention and Control, Beijing, China; 2 School of Public Health and Community Medicine, University of New South Wales, Sydney, NSW, Australia; 3 School of Electronic Engineering, University of Electronic Science and Technology of China, Chengdu, China

## Abstract

Serfling-type periodic regression models have been widely used to identify and analyse epidemic of influenza. In these approaches, the baseline is traditionally determined using cleaned historical non-epidemic data. However, we found that the previous exclusion of epidemic seasons was empirical, since year-year variations in the seasonal pattern of activity had been ignored. Therefore, excluding fixed ‘epidemic’ months did not seem reasonable. We made some adjustments in the rule of epidemic-period removal to avoid potentially subjective definition of the start and end of epidemic periods. We fitted the baseline iteratively. Firstly, we established a Serfling regression model based on the actual observations without any removals. After that, instead of manually excluding a predefined ‘epidemic’ period (the traditional method), we excluded observations which exceeded a calculated boundary. We then established Serfling regression once more using the cleaned data and excluded observations which exceeded a calculated boundary. We repeated this process until the R^2^ value stopped to increase. In addition, the definitions of the onset of influenza epidemic were heterogeneous, which might make it impossible to accurately evaluate the performance of alternative approaches. We then used this modified model to detect the peak timing of influenza instead of the onset of epidemic and compared this model with traditional Serfling models using observed weekly case counts of influenza-like illness (ILIs), in terms of sensitivity, specificity and lead time. A better performance was observed. In summary, we provide an adjusted Serfling model which may have improved performance over traditional models in early warning at arrival of peak timing of influenza.

## Introduction

Influenza has been a constant global health concern since the pandemic of 1918. Early detection of influenza events can help prioritise the allocation of public health resources and the planning of control measures. This highlights the need for optimal surveillance systems for public health control of emerging epidemics. Since September 2007, a surveillance system for influenza-like illness (ILI) and laboratory confirmed influenza has been conducted in Beijing, China [1]. The system has the potential to provide timely analysis and early detection of influenza events. Early warning at the start of the annual period of epidemic season has been documented in a number of previous studies [1–7]. The alarm is usually triggered if the observed number of events falls outside a calculated boundary. Forecasting the peak of influenza activity could inform decisions on the timing of vaccination campaigns and assist with stockpiling of influenza or planning of additional hospital bed capacity to meet high seasonal demand [6].

A set of approaches for early warning at the start of epidemic have been developed. Autoregressive Integrated Moving Average (ARIMA) model proposed by Box-Jenkins is the most classic method using long time series data [8]. In this model, method for smoothing a raw time series is often required. Cumulative sum (CUSUM) originally used in quality control, was used widely for early detection of the onset of epidemic [9,10]. However, the sample estimate of mean of CUSUM does not adjust for seasonality of the baseline. Mathematical model of transmission dynamics susceptible—infected—recovered-susceptible (SIRS) is growing used in forecast of the timing, duration and intensity of infectious disease [6,11,12]. Due to its inherent assumptions and complexity, this model has not been so widely used as Serfling regression model. Serfling regression model originally proposed by Serfling [13] has been widely used for influenza baseline establishment, epidemic detection and disease burden estimation using cleaned historical data. Related regression methods have been used in France, the UK CDSC, and ISIS in the Netherlands [14]. These harmonic models assume that the seasonal pattern of influenza activity during all non-epidemic periods remains stationary from year to year. Common practice is to exclude predefined epidemic periods based on long-term historical surveillance data to prevent the baseline from being raised by the epidemics, usually from the fall, winter and spring months (November or December through April) [15,16]. Given strong year-year variations in the seasonal pattern of activity, a simple fixed removal of epidemic-periods seems to be empirical and is likely to over- or under-estimation of the baseline [17]. It is therefore important to make some adjustments in the rule of epidemic-period removal to avoid potentially subjective definition of the start and end of epidemic periods [17]. Our study aims to use this adjusted Serfling model to improve the performance of early detection of influenza events over traditional (non-adjusted) models. To evaluate the performance of the adjusted Serfling regression model, we need to compare this model with traditional (non-adjusted) models. However, it can be challenging to evaluate the performance of these approaches, since the gold standard varies in different studies [18]. The definitions of the onset of influenza epidemic were heterogeneous, which may add to these difficulties in evaluating the performance of these approaches. Yang P *et al* used 40% of the highest weekly isolation rate as the onset of the epidemic [1]. Cowling BJ *et al* used 30% of the maximum level of isolation rate as the onset of epidemic season [4]. The week with the highest proportion of positive influenza isolations each season was commonly considered as the annual peak week of influenza season. The gold standard of peak timing of influenza activity can be defined more accurately and objectively than the onset of influenza epidemic, which allow accurate and reliable evaluation of the performance of alternative approaches. The starting of influenza epidemic is kind of a signal which warns at the arrival of influenza peak. When the epidemic started, there would be a sharp increase in the case numbers. And then the case number would peak soon. Therefore, in this study we compared the performance of the adjusted model with traditional Serfling models in the peak timing prediction of influenza. This may facilitate a more accurate evaluation of the performance of alternative approaches.

To our knowledge this is the first time to make adjustments in the rule of epidemic-period exclusion of Serfling-type regression to improved performance over traditional models in early detection of the peak timing of influenza.

## Materials and Methods

### Source of Data

Influenza surveillance in Beijing was established in 2007, and included a network of outpatient and emergency clinics of internal medicine and pediatric wards in 421 hospitals in Beijing. Participating referral doctors were required to diagnose ILI by using a strict ILIs definition (fever >38°C, either cough or sore throat) and to record the number of ILIs consultations by age group on a fixed form daily. These data were entered daily into the Beijing Monitoring and Early Warning System for Infectious Diseases in hospitals by designated hospital staff. Virological surveillance in a sub-group of 23 hospitals was also launched in 2007, where patients with ILI were tested for influenza within the same surveillance system. A total of 17 collaborating laboratories received specimens from 23 sentinel hospitals, and reported the weekly positive rates of influenza by type and subtype [1]. Pharyngeal swab specimens from the ILIs case-patients (within 3 days of symptom onset from patients who had not received antiviral drugs) were collected by designated staff. The specimens were transported to the correspondent laboratories in viral transport medium at 4°C, for subsequent isolation and identification. Weekly type- and subtype- specific positive rates of influenza were reported by collaborating laboratories reported the [1]. This allows direct calculation of weekly influenza positive rates. This surveillance was originally designed to be active in the winter season, but after pandemic H1N1 2009, was extended to operate year-round. The observed weekly case counts of ILI from September 2007 to July 2014 in Beijing were obtained from this surveillance system and these data were used to establish and test the Serfling regression (S1 Dataset).

The peak timing of influenza was determined from laboratory data of confirmed influenza isolates. The annual highest proportion of positive influenza isolations each season was considered as the annual peak of seasonal influenza activity, and used as a gold standard for evaluating the performance of our forecasts.

### Statistical Analysis

**Fig 1:**
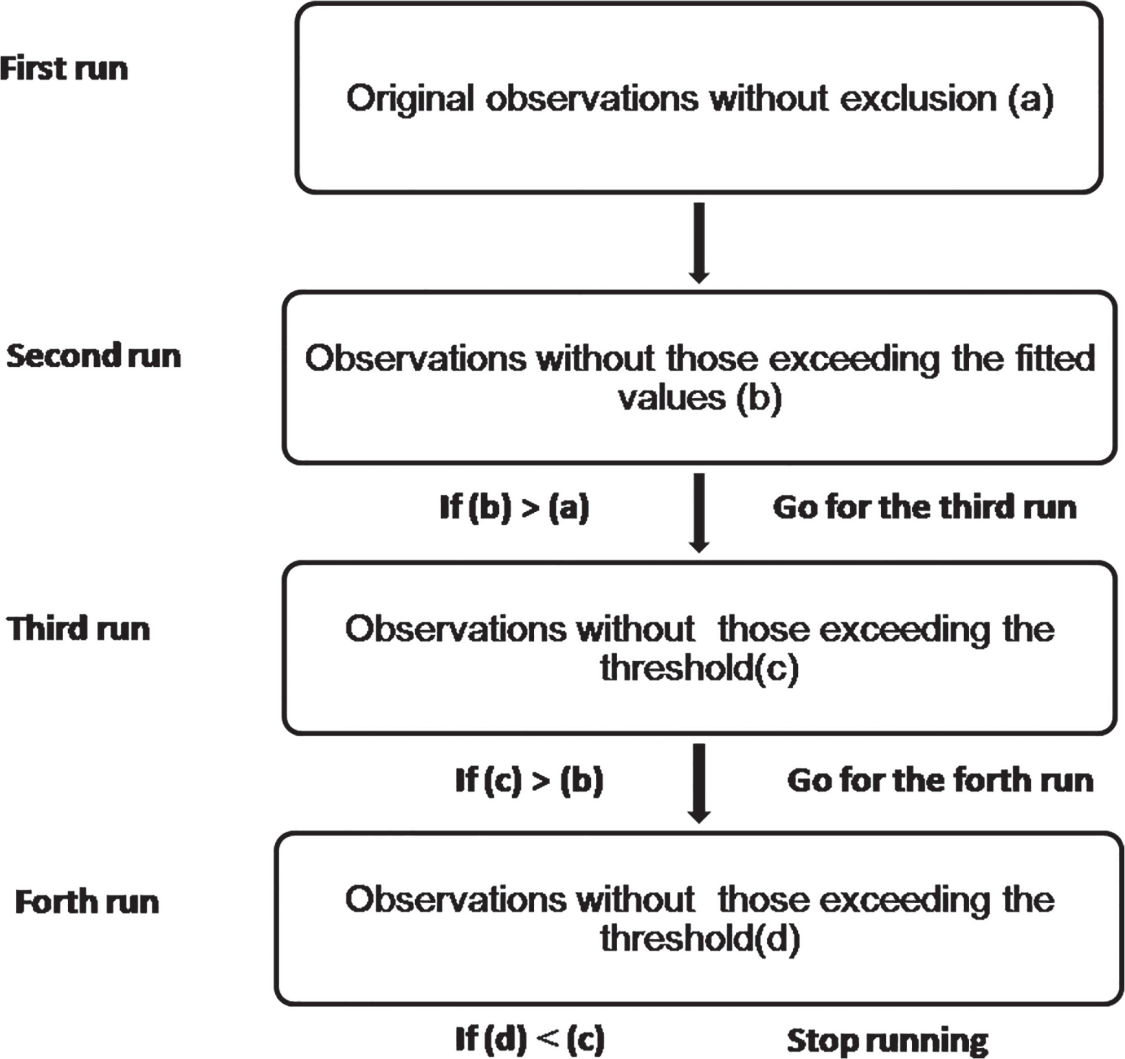
Schematic diagram of the adjusted Serfling regression model. Note: (a) R^2^ value in the first run. (b) R^2^ value in the second run. (c) R^2^ value in the third run. (d) R^2^ value in the fourth run.

In this study, we made the following modifications to the exclusion or removal of epidemic months of influenza. Due to the lack of accurately pre-specified baseline case numbers and the number of cases in epidemic, we calculated the weekly baseline case number of ILIs iteratively, starting with the observed number of ILIs counts (Fig. 1). In the first run, we established a Serfling regression model based on the actual weekly observed ILI counts without any exclusion of historical data. The model structure is defined as follows [19]:

$$Y_{t}=\beta_{0}+\beta_{1}t+\beta_{2}t^{2}+\beta_{3}\;\text{sin}\left(2\pi t/52.18\right)+\beta_{4}\;\text{cos}\left(2\pi t/52.18\right)+\beta_{5}\;\text{sin}\left(2\pi t/26.09\right)+\beta_{6}\;\text{cos}\left(2\pi t/26.09\right)+\varepsilon_{t}$$ (1)

Where Y_t_ is the number of ILIs reported in week t; β_1_, β_2_,β_3_,…β_6_ are regression coefficients to be estimated; and ε_t_ is a normally distributed error term. The R^2^ was used as the measure for goodness of fit in our model.

After that, instead of manually excluding a predefined ‘epidemic’ period (the traditional method), we excluded the actual observations which exceeded the fitted values from the first round of regression. We then established Serfling regression once more using cleaned data as new baseline data to construct a 95% forecast interval and if one observed ILIs count in a given week exceeded the threshold then the observation would be excluded from baseline data. Since the second round of regression, the upper prediction limit of the baseline level forecasted by the model had been set as the threshold. We repeated this process until the R^2^ value stopped to increase. The upper limit of the fitted baseline with the highest R^2^ value was treated as the threshold of the influenza peak season. The start of peak activity was defined when the threshold was exceeded for two or more consecutive weeks.

For each run, we compared observed count with the calculated threshold from each round of regression and got a new baseline data to construct a new threshold. Iteration involves making an initial estimate of the parameter values. The initial parameter estimates should be based on prior experience of the data or a sensible guess based on knowledge of the function used to fit the data [20]. Since the actual observations include both baseline data and epidemics, the baseline level constructed from the first round regression without exclusion of epidemic periods was raised. It was unknown that to what extent the baseline level was raised. Using the 95% upper prediction limit as the threshold seemed not rational. After the initial regression, we set the fitted values from the first round of regression empirically as the threshold to roughly exclude probable epidemics. Baseline data was not static. Data considered as baseline data in this run might be excluded in the next round of regression. Similarly, data excluded from baseline data might be included in again.

To check if the variation of traditional harmonic approach is technically sound, we simulated weekly ILIs observations by adding simulated epidemics to predefined baseline. The baseline was established by Serfling regression (formula 1), using all actual observed weekly ILIs counts without any exclusion of historical data. Based on the locations of Beijing and the range in epidemic periods described in previous literature [1,21,22], seven epidemic periods of influenza were defined (weeks 41–14, 45–14, 45–9, 45–5, 49–5, 49–52, and 1–5). The magnitude of each simulated epidemic was defined at 10% or 20% of the actually reported weekly ILI counts during defined epidemic periods. There were a total of 98 combinations of epidemics in seven influenza seasons. Each season, we randomly selected one epidemic adding into the estimated baseline. The onset of an epidemic was randomly selected from week 41 to 1 of each season. In order to reduce sampling errors, we repeated the sampling five times. The adjusted Serfling regression model was then applied to detect the predefined baseline and epidemics, using these five simulations.

To examine the efficiency of our adjustment in the rule of exclusion of epidemic seasons, we compared the performance with the traditional manual removal (traditional Serfling model) in peak forecasting, using metrics of sensitivity, specificity, and timeliness. Influenza peak was defined as detected when two consecutive signals were triggered ahead of the true peak timing of influenza virus activity within the same influenza season (from September to April). Sensitivity was defined as the proportion of successful peak timings detected. Specificity was defined as (1-r/m), where r was the number of false positive alarms and m was the total number of non-peak weeks. Timeliness was defined as the time (average number of weeks) ahead of the true peak at which a signal was detected. The most desirable method might have maximum sensitivity and specificity, and timeliness. Due to lowering the specificity using a different threshold for peak detection, a higher sensitivity and timeliness might be achieved. We set specificities of traditional models at the same level or even lower, and compared the sensitivity and timeliness of the adjusted model with traditional models.

Actual surveillance data from September 2007 to July 2014 were firstly used to test the ability of the models to detect the influenza peak retrospectively. After the test, we then conducted prospective predictions of annual peak timing of seasonal influenza in Beijing, using this adjusted Serfling regression model. Since Serfling-type regression model usually require three or more years of historical data, we predicted the peak timing of annual seasonal influenza from September 2010 (corresponding to week 37, 2010) to July 2014. From week 37, 2010, data from all preceding weeks was used to construct a 95% forecast interval and if the data for the current week exceeded the upper limit then an alert would be generated. We then used the data as of week 37, 2010 to refit the baseline and calculated a new threshold. If the observed ILI count of week 38, 2010 exceeded the threshold again, we might consider that it was a signal warning at the arrival of the annual peak timing of influenza activity. Similarly, the adjusted Serfling model had been refitted weekly to generate a new threshold since week 39, 2010.All statistical analyses were conducted in R, version 3.0.1 (R Foundation for Statistical Computing, Vienna, Austria).

## Results

### General Description

**Fig 2:**
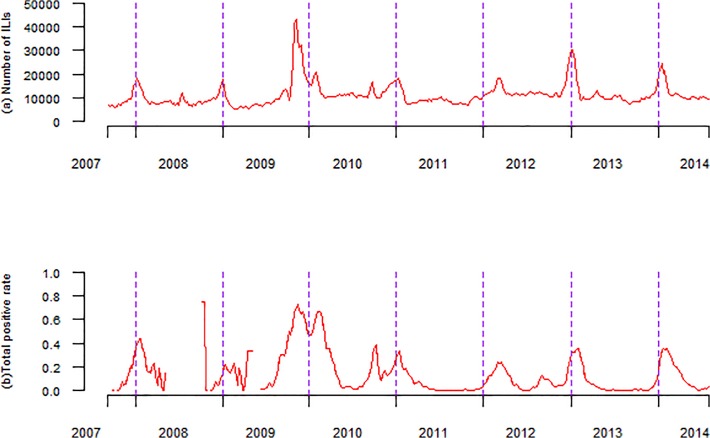
Weekly observed ILI counts, and weekly positive rate of influenza in Beijing, China, September 2007 to July 2014. Note: (a) Weekly observed ILI counts, reported in ILI surveillance system. (b) Weekly total positive rate of influenza isolates. The annual peak of positive rate was considered as a gold standard to determine annual peak of influenza season.

From September 2007 through July 2014, there were a total of 7 influenza seasons. Both the weekly ILI counts and weekly positive isolation rates showed apparent seasonality. Influenza peaks were highly concentrated in winter months, around week 4, corresponding to late January (Fig. 2, S1 Dataset). Semi-annual cycle was also observed in some of seven seasons. However, pandemic H1N1 2009 was quite different in both in the peak timing and the maximum level of influenza activity. During the influenza season (2009–2010), peak timing moved forward from December to November, and the peak level of positive isolation rate increased from an average of 23.1% in previous years to 72.8%. The peak timing moved forward further from November to October during the next influenza season (2010–2011), but the maximum level of positive rate decreased sharply to 38.1% (Table 1). As shown in Fig. 2, we found that the week of the maximum positive rate of influenza isolation was almost identical to that of the highest ILI counts (except during the 2010–2011 influenza season).

**Table 1:** The true peak timing of seven influenza seasons and corresponding maximum positive isolation rate of influenza virus.

Season	True peak week	Positive isolation rate (%)
2007–2008	Week 2 of 2008	43.6
2008–2009	Week 7 of 2009	23.1
2009–2010	Week 45 of 2009	72.8
2010–2011	Week 40 of 2010	38.1
2011–2012	Week 9 of 2012	24.5
2012–2013	Week 4 of 2013	35.4
2013–2014	Week 4 of 2014	40.7

Note: The annual highest proportion of positive influenza isolations was considered as the annual peak of influenza season.

### Simulations and Verification

**Fig 3:**
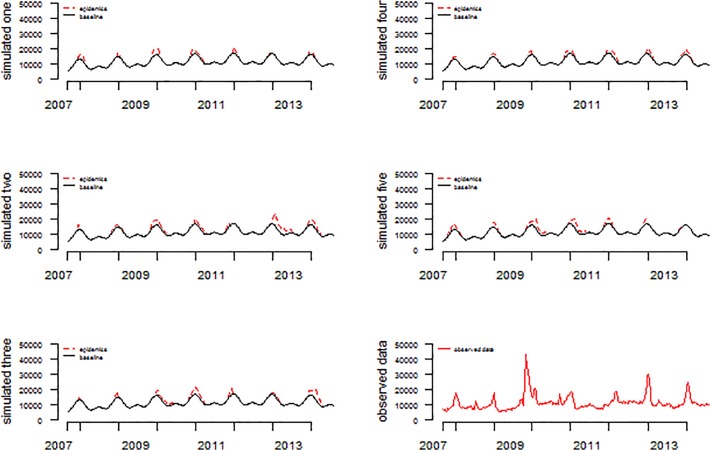
Five simulated data consisting of baseline data and simulated epidemics, and weekly actually observed ILI counts.

We simulated five different time series of weekly case counts of ILIs from September 2007 to July 2014(Fig. 3, S2, S3, S4, S5, and S6 Dataset). The magnitude of the epidemic was smaller than the actual epidemic level. The performance of the adjusted Serfling model was examined using these five simulated data. The iterative fitting method succeeded in detecting the baseline and epidemic through multiple rounds of iterative regressions. The R^2^ value reached 1 after 3 or 4 iterations and then decreased gradually. The baseline levels determined by the adjusted Serfling regression model were identical to predefined simulations. The onset, duration and the magnitude of each epidemic was accurately detected by this adjusted model.

### Retrospective Forecasts

Given its good performance in determine the baseline level and epidemics with five simulations, we then used this adjusted model to forecast the peak activity of influenza using historical ILIs surveillance data from 2007 to 2014.

**Fig 4:**
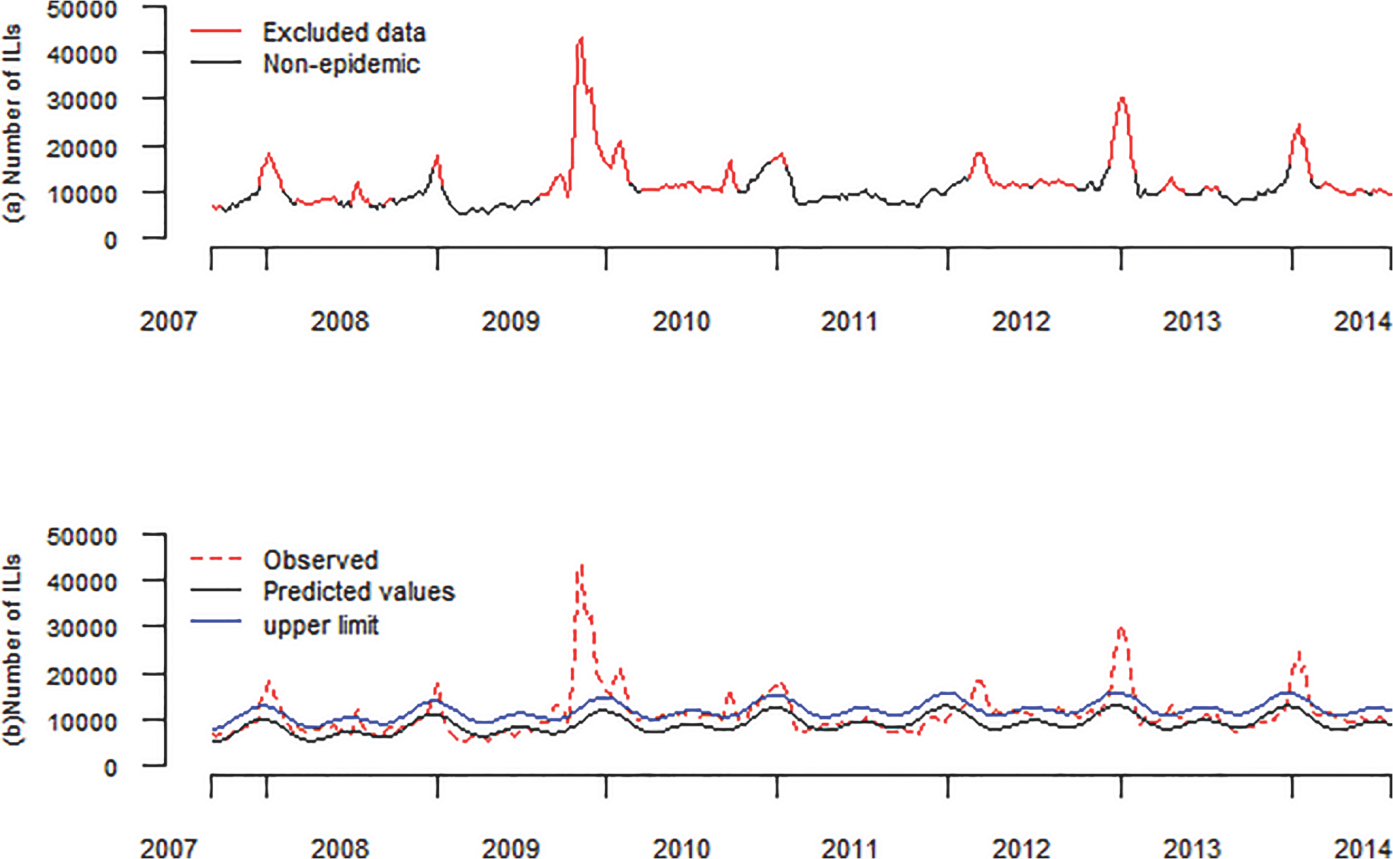
The results of retrospective forecasting, using the adjusted Serfling regression model. Note: (a) Excluded data based on the first-round Serfling regression model. (b) weekly observed ILI, reported in ILI surveillance system (red dotted line), weekly predicted ILI counts from the second round of Serfling regression model (black thinner line), weekly upper limit for ILI counts (blue line).

In the first run, the R^2^ was 0.2852.After excluding the data based on the first-round Serfling regression model (shown in Fig. 4). The R^2^ increased to 0.5934, and then decreased gradually. Thus baseline data, with which a baseline was fitted with a R^2^ of 0.5934, was finally defined.

Table 2 and Fig. 4 showed the retrospective forecasting of the adjusted Serfling model compared with traditional models. We found that the adjusted Serfling model far outperformed the traditional Serfling models both in sensitivity and average number of weeks ahead of true peaks. The specificity of adjusted Serfling model is relative lower than those traditional models, with a specificity of 97.8%. However, the highest sensitivity of traditional Serfling models was 57.1% (5/7). Unlike traditional models, the adjusted model showed sensitivity as high as 100% (7/7). Most of traditional models showed no more than three weeks lead-timing. However, the iterative fitting process showed an average lead-timing of 4.4 weeks.

**Table 2:** Results of retrospective analysis, compared with traditional Serfling models.

Methods	Sensitivity (%,n/N)	Specificity (%)	Average No. of weeks ahead	R^2^
Traditional 1 (week 41–14)	57.1 (4/7)	99.4	4.3	0.2615
Traditional 2 (week 45–14)	14.3 (1/7)	100.0	2.0	0.1547
Traditional 3 (week 45–9)	28.6 (2/7)	100.0	3.0	0.1531
Traditional 4 (week 45–5)	42.9 (3/7)	100.0	3.0	0.1422
Traditional 5 (week 49–5)	42.9 (3/7)	100.0	3.0	0.1689
Traditional 6 (week 49–52)	28.6 (2/7)	100.0	3.0	0.2571
Traditional 7 (week 1–5)	28.6 (2/7)	100.0	3.0	0.246
Adjusted Serfling model	100.0 (7/7)	97.8	4.4	0.5934

Note: Traditional 1 (week 41–14) refers to establish baseline after excluding annual week 41 to week 14, using Serfling regression model. Similarly, traditional 2 (week 45–14) refers to establish the baseline after the removal of annual week 45 to week 14, using Serfling regression.

When we used 20% upper prediction limits to decrease specificities and increase sensitivities of seven traditional models (Table 3). We found that when traditional models had lower specificities, their sensitivities were still lower that the adjusted model. All the seven models failed to detect the coming of peak during the season of 2008 to 2009. Regarding the timeliness, 5/7 of traditional models showed smaller number of weeks ahead of the true peak timing of influenza activity.

**Table 3:** Results of retrospective analysis, compared with traditional Serfling models with a lower threshold (20% upper prediction limits).

Methods	Sensitivity (%,n/N)	Specificity (%)	Average No. of weeks ahead
Traditional 1 (week 41–14)	85.7(6/7)	95.6	4.7
Traditional 2 (week 45–14)	42.9 (3/7)	96.3	6.0
Traditional 3 (week 45–9)	85.7 (6/7)	96.6	3.7
Traditional 4 (week 45–5)	85.7 (6/7)	96.0	4.2
Traditional 5 (week 49–5)	85.7 (6/7)	97.2	4.2
Traditional 6 (week 49–52)	85.7 (6/7)	96.9	3.8
Traditional 7 (week 1–5)	85.7 (6/7)	96.6	3.8
Adjusted Serfling model	100.0 (7/7)	97.8	4.4

### Prospective Forecasts

**Fig 5:**
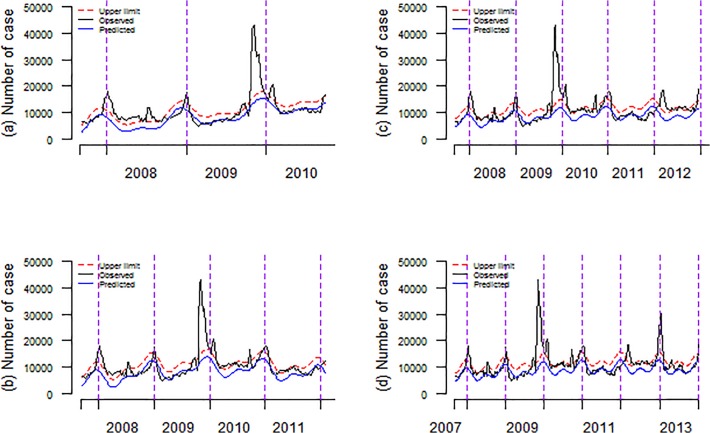
The results of prospective forecasting, using the adjusted Serfling regression model. Note: (a) Prospective prediction of the arrival of peak activity of the 2010–2011 influenza season. (b) Prospective prediction of the arrival of peak activity of the 2011–2012 influenza season. (c) Prospective prediction of the arrival of peak activity of the 2012–2013 influenza season. (d) Prospective prediction of the arrival of peak activity of the 2013–2014 influenza season.

Table 4 and Fig. 5 showed that the prospective predicting of peak timing of influenza. Similarly, the adjusted Serfling model far outperformed the traditional Serfling models both in sensitivity and timeliness. The adjusted model succeeded in early warning at the arrival of each peak of influenza from influenza season 2010–2011 to 2013–2014. Unlike the adjusted model, all seven traditional models failed to detect the coming of peak during the season of 2010 to 2011. The highest sensitivities of traditional models were 75% (3/4). All these traditional models failed to generate alert warning at the arrival of peak activity of influenza season 2010–2011. Traditional models excluding periods of week 45–9 and week 49–52 had also failed to issue alert warning at the coming of peak during the season of 2013–2014. What worst is the traditional model excluding annual data from the 45^th^ week to the 14^th^ week had detected no signal forecasting the arrival of seven influenza peaks.

**Table 4:** Results of prospective prediction, compared with traditional Serfling models.

Methods	Sensitivity (%,n/N)	Specificity (%)	Average No. of week ahead
Traditional 1 (week 41–14)	75.0 (3/4)	99.3	2.3
Traditional 2 (week 45–14)	0 (0/4)	*-	*-
Traditional 3 (week 45–9)	50.0 (2/4)	100.0	2.0
Traditional 4 (week 45–5)	50.0 (2/4)	100.0	3.5
Traditional 5 (week 49–5)	75.0 (3/4)	100.0	2.3
Traditional 6 (week 49–52)	50.0 (2/4)	100.0	2.0
Traditional 7 (week 1–5)	75.0 (3/4)	100.0	2.0
Adjusted Serfling model	100.0 (4/4)	97.6	4.5

* Since those models failed to predict the peak of annual influenza season, the specificity and lead time were not available.

Regarding the timeliness, the adjusted Serfling model showed more lead-timing, with an average of 4.5 weeks before the actual peak timing. Using the actual observed weekly ILIs counts as of week 37 of 2010, one signal was triggered by the adjusted model triggered. By the same way, using surveillance data as week 38 of 2010, one more signal was triggered. Two consecutive signals were considered as alert signal warning at the arrival of peak activity. The highest positive rate of influenza was then observed in week 40 of 2010. It showed that the adjusted model should forecast the coming of the peak influenza prospectively. Similarly, as of week 50 of 2012, two consecutive alarm signals were given, indicating the coming of influenza peak. These alerts were proved to be triggered 7 weeks before the peak of positive isolation rate of influenza virus. Compared with traditional models, the adjusted model had relative lower specificity, with a specificity of 97.6%.

## Discussions

Serfling regression models have been used since the mid-1960s used to determine epidemic influenza activity and excess mortality attributed to influenza [13,19,22]. However, estimation of the seasonal baseline is a challenging statistical problem. While the observations consist of baseline data and epidemic values, the epidemic is often hard to be identified and excluded. On this basis, excluding the influence of epidemic activity is a requirement of Serfling regression models to estimate the baseline level [23]. Beijing is located in temperate region, where influenza typically peaks seasonally in the end of December or in the first of January [1,24]. However, great changes had been taken in the seasonal pattern of influenza in Beijing, due to pandemic H1N1 2009 virus, which caused the first pandemic of the 21st century. The timing of the peak moved forward to October, with a magnitude 4 times greater compared with the same period in the previous year (2008–2009).Under these circumstances, establishment of a seasonal baseline using traditional Serfling regression which excludes predefined “epidemic periods” [22,25–28] seems unreasonalble. It is therefore important to make some adjustments in the rule of epidemic-period removal to increase the flexibility of Serfling-type periodic regression. To address this challenge, we modified the rule of removal of epidemic months. In this study, we used and variation of traditional Serfling regression model instead of the more rigid exclusion of pre-specified epidemic periods. We excluded some traditionally defined baseline periods and also included part of the traditionally defined epidemic periods, which was quite different from traditional practice. The deviation between the observed and expected values was so slight that it was not easy to be detected by empirical observation. However, this adjusted model could detect more slight shifts from the threshold in traditionally defined baseline periods, since the exclusion was decided by the upper limits from multiple rounds of iterative calculation rather than dependent on the predefined epidemic periods. Similarly, the adjusted model could find some observations were not high enough to be removed in the traditionally defined epidemic periods.

We aimed to evaluate the performance of different methods for exclusion of the “epidemic period” and to identify a more optimal method. Compared with the traditional exclusion of epidemic periods, our adjusted method showed higher sensitivity and timeliness both in retrospective and prospective forecasts. Some of the traditional models showed higher specificity. However, when traditional models had lower specificities, using 20% upper prediction limits as the threshold, their sensitivities were still lower than the adjusted model. In addition, we also found that all the seven models failed to detect the coming of peak during the season of 2008 to 2009. This might be the result of not considering the potential influence from the emergency of pandemic H1N1 2009. During the pandemic H1N1 2009, large variations had been observed in the timing of the peak and the magnitude. Excluding traditionally defined epidemic periods might have raised the baseline level and thus decreased the sensitivity of these models. For the purpose of identifying and predicting the peak of influenza activity, high sensitivity is the most important characteristic and is often the priority which we should put on. Our adjusted model is therefore outperformed the traditional Serfling models in early warning at the arrival of annual peak influenza activity. Results showed that our adjusted model could generate a signal 4–5 weeks ahead of the true peak. However, in real word, the model actually could trigger an alert 6–9 (2–4 more) weeks ahead of the time when we realize the arrival of the true peak. Firstly, it usually took 1–2weeks to get the positive rate of influenza from laboratory testing. Secondly, it took at least one week to determine whether the positive rate peaked. Only the positive rate started to decrease, the peak of positive rate could be detected. It might take one more week if decrease in positive rate of influenza for two consecutive weeks was needed to define the peak week of influenza activity. What’s more, it would take more weeks to determine the arrival of peak if the positive rate of influenza increased again after temporary decrease. This may facilitate timely initiation of precautionary measures for influenza activity. Forecasting the peak of influenza activity could inform decisions on the timing of vaccination campaigns or other control measures for influenza [29]. Administering vaccines in the early stages of an epidemic is more effective than starting vaccination after the epidemic peak [7,29]. Peak forecasting could also assist with stockpiling of influenza or planning of additional hospital bed capacity to meet high seasonal demand [6]. Our flexible exclusion of epidemic periods might facilitate the baseline establishment and correspondingly be helpful in assessing the influenza-associated morbidity or mortality during epidemic periods. There are some limitations in this study. First, virologic surveillance for influenza was originally half-year round in Beijing, and had been expanded to year-round since the pandemic H1N1 2009. The variation in the coverage of this surveillance might have the potential to affect the consistency in the laboratory testing method or sampling method, which might have impact on performance evaluation of alternative methods. Second, the adjusted Serfling regression described in the study could be improved if more variables, such as meteorological data, were incorporated. Third, there was only seven influenza seasons in this study, and influenza is subject to seasonal variation in epidemiology. To address this challenge, simulations of influenza seasons could be an alternative method. However, it is difficult to evaluate how close these simulations come to the actual influenza season. In time, longer time series will improve the accuracy and reliability of evaluation on the performance of peak forecasting methods.

## Conclusions

In summary, the adjusted Serfling model has the potential to improve the performance of early warning at the arrival of peak activity of influenza. The high performance of the adjusted model was not limited to retrospective analysis, but it exhibited good performance in prospective forecasting of the peak of influenza. Due to its simplicity and good performance in prospective forecasting, we suggest that the adjusted model can be generalized in the field of early detection of influenza events.

## Supporting Information

S1 Dataset Raw data of weekly ILI counts and positive rate from September 2007 through July 2014. (XLS)

S2 Dataset Simulated data one. (XLS)

S3 Dataset Simulated data two. (XLS)

S4 Dataset Simulated data three. (XLS)

S5 Dataset Simulated data four. (XLS)

S6 Dataset Simulated data five. (XLS)
